# Flap necrosis after palatoplasty in irradiated patient and its reconstruction with tunnelized-facial artery myomucosal island flap

**DOI:** 10.1186/s40902-017-0121-5

**Published:** 2017-08-25

**Authors:** Hye-In Jeong, Hye-Min Cho, Jongyeol Park, Yong Hoon Cha, Hyung Jun Kim, Woong Nam

**Affiliations:** Department of Oral and Maxillofacial Surgery, Yonsei University, College of Dentistry, Seoul, South Korea

## Abstract

**Background:**

Tunneled transposition of the facial artery myomucosal (FAMM) island flap on the lingual side of the mandible has been reported for intraoral as well as oropharyngeal reconstruction. This modified technique overcomes the limitations of short range and dentition and further confirms the flexibility of the flap. This paper presents a case of reconstructing secondary soft palatal defect due to flap necrosis following two-flap palatoplasty in irradiated patient with lingually transposed facial artery myomucosal island flap.

**Case presentation:**

The authors successfully reconstructed secondary soft palatal defect due to flap necrosis following two-flap palatoplasty in an irradiated 59-year-old female patient with tunnelized-facial artery myomucosal island flap (t-FAMMIF).

**Conclusions:**

Islanding and tunneling modification extends the versatility of the FAMM flap in the reconstruction of soft palatal defects post tumor excision and even after radiation, giving a great range of rotation and eliminating the need for revision in a second stage procedure. The authors thus highly recommend this versatile flap for the reconstruction of small and medium-sized oral defects.

## Background

Depending on the site and size of the defect, fasciocutaneous free flaps [1–4], locoregional pedicled flaps [5, 6], and local flaps [7–9] can be used to reconstruct soft palatal defects following tumor resection to prevent nasal speech with excessive air escape and nasal regurgitation of food. Among these, buccinator-based myomucosal or facial artery myomucosal (FAMM) flaps are rich in blood supply, have appropriate thickness and considerable mucosal paddle [10], and can secrete saliva; hence, they are good choices for the repair of intraoral medium-sized mucosal defects [11].

Pribaz et al. described the many advantages of the FAMM flap over flaps based on the buccal artery, including the greater versatility in reconstructing a wide range of difficult intraoral problems for which conventional techniques have failed [12]. The FAMM island flap was recently popularized by Zhao et al., who also described a myomucosal island flap (BUMIF, buccinator myomucosal island flap) for use in cases of cleft palate and periorbital defects [13]. As a disadvantage of these flaps, shortage of range may occur when covering contralateral defects in the floor of the mouth and gingiva, particularly in dentate patients. Tunneled transposition of the FAMM island flap on the lingual side of the mandible has been reported for intraoral as well as oropharyngeal reconstruction. This technique overcomes the limitations of short range and dentition and further confirms the flexibility of the flap [7, 8, 14–16]. We used this flap for the first time in 2013 for reconstruction of palatomaxillary defect [17]. This paper presents another case of reconstructing secondary soft palatal defect due to flap necrosis following two-flap palatoplasty in irradiated patient with a lingually transposed facial artery myomucosal island flap.

## Case presentation

**Fig. 1:**
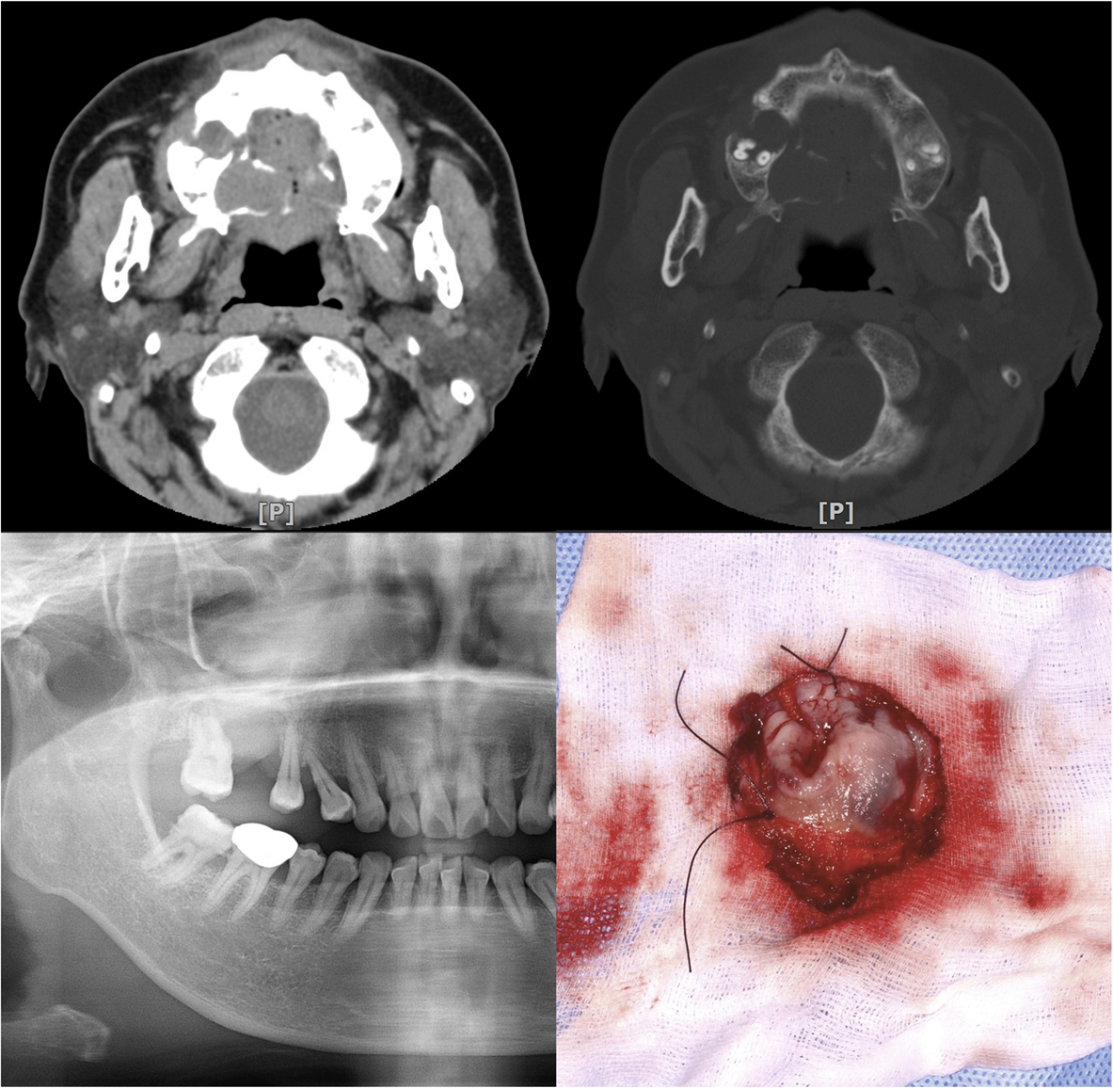
Preoperative computerized tomographic (CT) scan (*top*) and panoramic radiograph (*bottom left*) showed a round mass on the right palatal area. Postoperatively excised mass (*bottom right*)

**Fig. 2:**
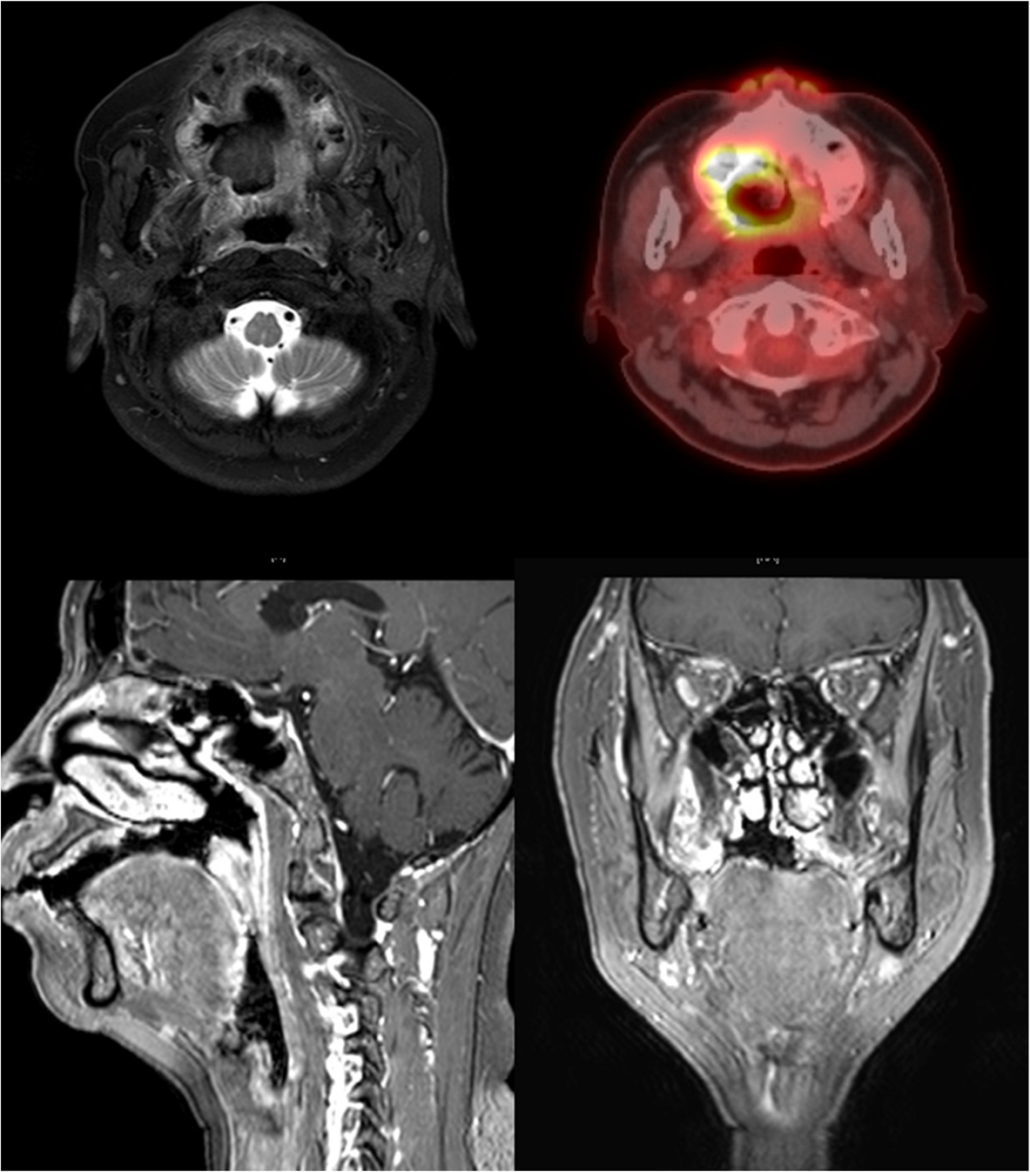
Magnetic resonance imaging (*top left* and *bottom*) and whole-body positron emission tomography (*top right*) showed no unusual finding following operation and mildly increased FDG uptake on the right hard palate, likely postoperative changes. There was no evidence of distant metastasis

A 59-year-old female patient visited our oral and maxillofacial department clinic complaining of a sense of discomfort in the right posterior palatal area. The patient did not remember exactly when the symptom began. The patient had no other concerned medical history. On clinical examination, a dome-shaped mass of 2.0 × 2.5 × 1.0 cm with clear border and no ulceration was observed in the right posterior palatal area. On the next days of admission, incisional biopsy was performed under local anesthesia. Pleomorphic adenoma (with central coagulative necrosis, most likely traumatized pleomorphic adenoma) was reported histopathologically. Hence, the patient underwent simple mass excision with safety margin under general anesthesia without any additional examination (Fig. 1). Postoperative histopathologic report was epithelial myoepithelial carcinoma with positive basal resection margin. Magnetic resonance imaging of the head and neck and whole-body positron emission tomography were performed for further examination, but there was no evidence of distant metastasis (pT2N0M0, stage II) that was shown (Fig. 2). Postoperative radiation therapy was administered to the primary site at the Department of Radiation Oncology, and the total radiation dose was 6148, 5400, and 4500 cGy at the operation site, border area, and lateral cervical lymph node level IB and II, respectively, for 39 days. There were no significant complications other than oral mucositis.

**Fig. 3:**
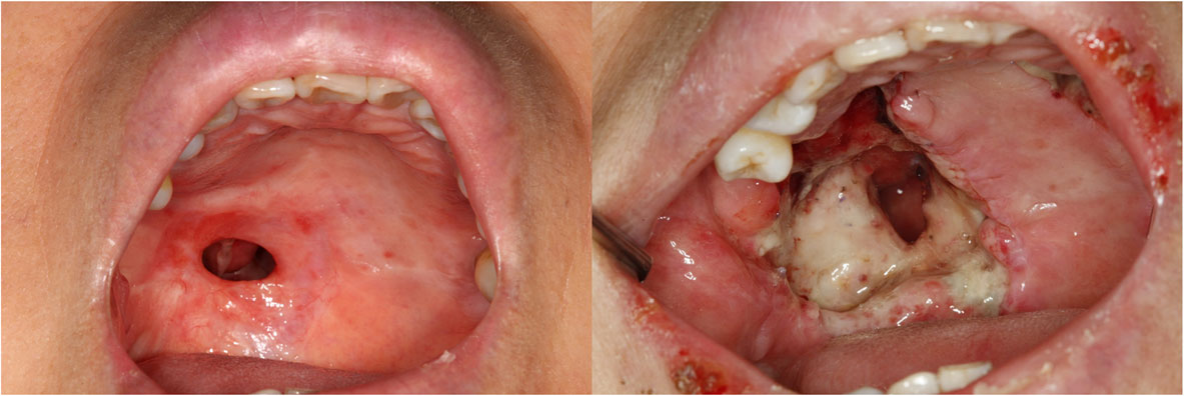
A 1.5 × 1.0 cm fistula formation after radiation therapy (*left*) and flap necrosis after palatoplasty (*right*)

**Fig. 4:**
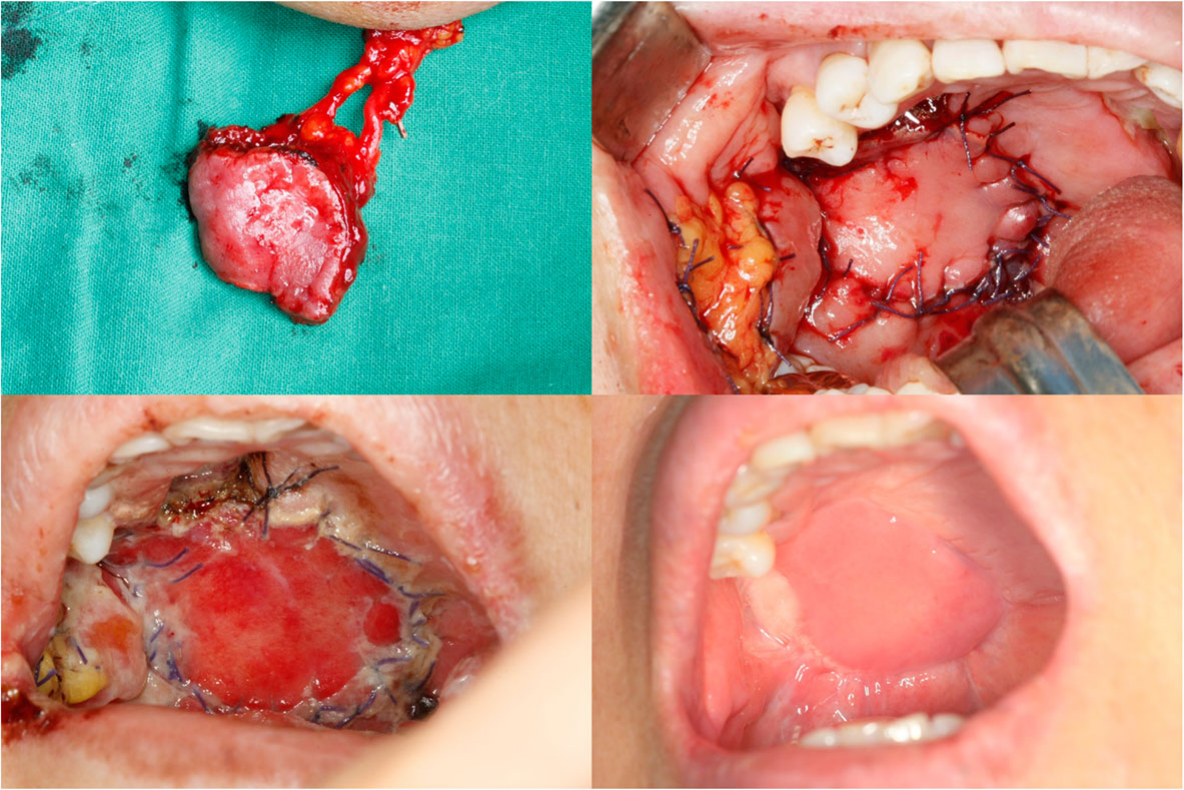
Extraorally transposed t-FAMMIF (*top left*). Palatal defect was closed with t-FAMMIF, and donor site was restored using buccal fat pad flap (*top right*). Postoperative 1 day (*bottom left*) and 3 months (*bottom right*)

After radiation therapy, a 1.5 × 1.0 cm fistula occurred in the right site, which was the operated site, and a fistula closure was done using two-flap palatoplasty under general anesthesia at 8 months after radiation therapy. However, the operated right side flap, which had poor blood circulation after radiation therapy, was necrotized (Fig. 3). We removed the necrotized flap under general anesthesia and designed a facial artery myomucosal island flap containing the right mucosal membrane and buccinators, using the facial artery as a trophic blood supply to the flap. The flap was transposed by tunneling to restore the defect through the lingual side of the mandible. The donor was restored using the ipsilateral buccal fat pad flap. After the operation, the nasal and oral opening was closed and properly healed up (Fig. 4).

## Conclusions

Reconstruction of maxillofacial defects lets surgeon find the most satisfactory flap both esthetically and functionally. It requires not just a knowledge of the flap, but an ability to think and plan in three dimensions [18]. In particular, it is physiologically optimal and advantageous to reconstruct oral mucosa with the same kind of tissue [19]. Though microsurgery has advanced greatly, the morbidity of the donor site, extended surgery, and longer hospitalization constitute limitations when applying this surgical method to patients with poor health. Thus, the defect, when smaller than 8–10 cm, can be reconstructed properly with local or locoregional flaps [20].

Since it was introduced by Janusz Bardach in 1967, two-flap palatoplasty remains a highly successful technique for closure of a variety of palatal clefts, with low fistula incidence [21] and yielding excellent surgical and speech outcomes [22]. We therefore decided to apply this technique to closing the fistula with the consent of the patient although the patient had had postoperative radiotherapy. However, poor blood circulation in the right descending palatal artery intraoperatively eventually led to the right palatal flap becoming necrotized. When deciding the next relief surgery, we considered free flap (radial forearm) or local flap (FAMM flap) and chose local flap on the principle of replacing like with like [9]. In contrast to reconstruction with the FAMM flap, which has traditionally been described as a two-stage procedure [23], this modification by tunneling on the lingual side of the mandible made the operation more simple and versatile [7, 23, 24].

The facial artery was easily identified and preserved with a Doppler probe. Without a 2-team approach, the flap was easily harvested and tunneled submandibularly on the lingual side of the mandible and finally transposed to the defect site and sutured. In Fig. 4 (bottom left), the flap showed some degree of venous congestion immediate postoperatively, but became resolved in a few days with adequate venous drainage provided by submucosal plexus [13]. The donor site was covered with buccal fat pad advancement. As seen in Fig. 4 (bottom right), the flap shows an excellent color match with recipient tissue.

**Fig. 5:**
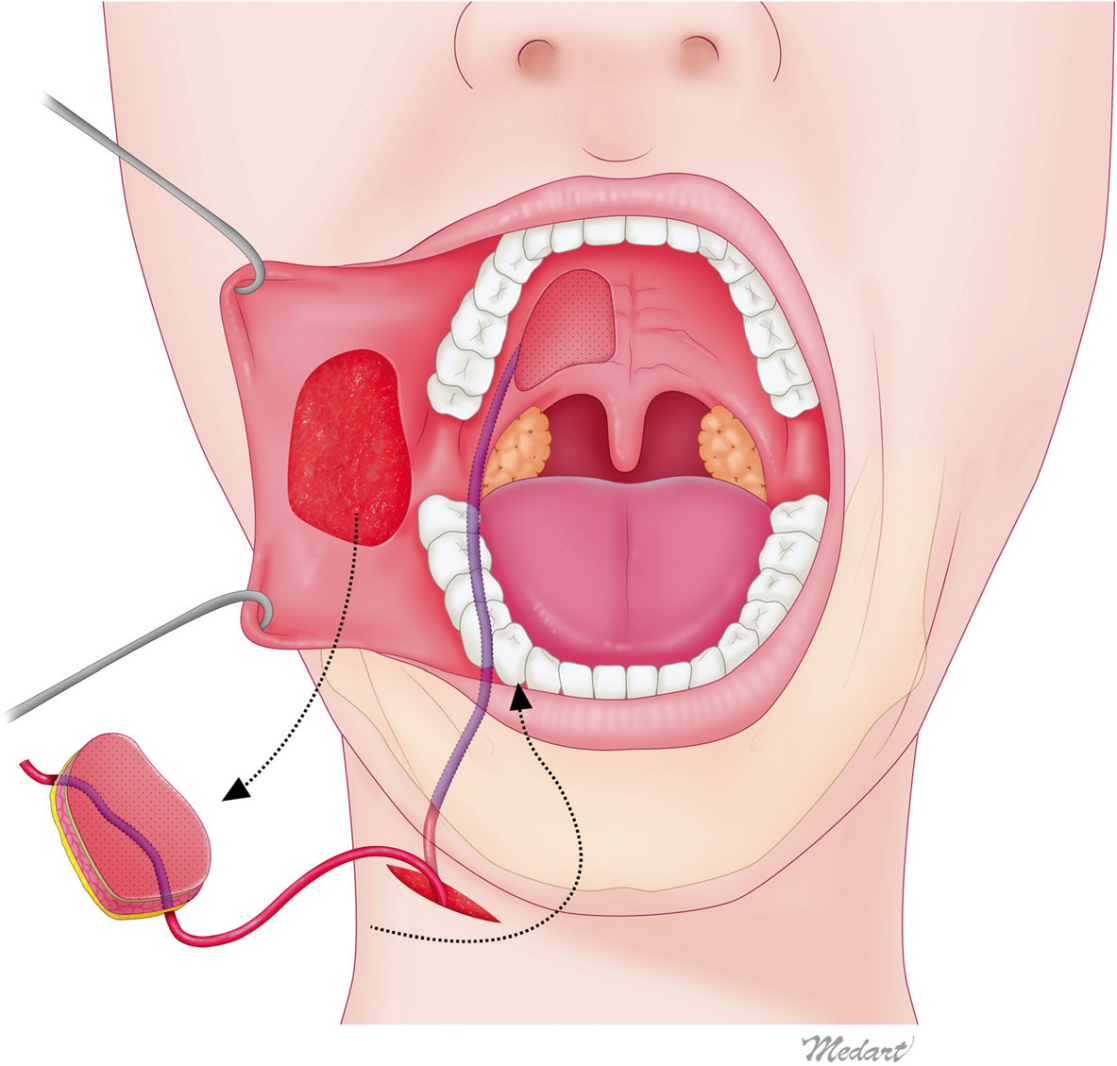
Schematic image of this flap surgery

This flap provides an abundant source of local tissue like buccinator muscle and may be reinnervated by the recipient site motor nerve, and the mucosa with connective and glandular tissue, which retains the secretory function of the native soft palate [9]. It is also advantageous that the flap is hairless and more pliable than a skin flap. It is known that no radiotherapy-associated shrinking has been observed [9]. The following are some basic precautions: first, care must be taken to preserve the Stensen duct, the orifice of which must be identified and preserved during flap harvesting. Second, avoid damage to the marginal mandibular branch of the facial nerve during tunneling. Identify the course of the nerve with a nerve stimulator. Third, confirm if there is a neck lymph node metastasis because facial vessel dissection may impair the oncologic safety (the presence of lymph node metastasis is a contraindication for the use of this flap). Fourth, check the postoperative mouth opening. Trismus may occur as a result of buccinator harvesting, but can be avoided with active postoperative mouth opening exercise. Finally, although there are several terms for buccinator‑based myomucosal flaps such as Bozola flap, Zhao flap, FAMM flap, BUMMIF, myomucosal cheek flap, buccal musculomucosal flap, buccal mucosal transposition flap, and intraoral cheek transposition flap, the author coined the term “tunnelized-Facial Myo-Mucosal Island Flap (t-FAMMIF)” because this modified flap is meant to be used for more innovative purposes than traditional methods are intended (Fig. 5) [9]. The authors highly recommend this flap to oral and maxillofacial surgeons who treat oral cancer patients as it may be widely used in reconstruction during initial stages of oral cancer due to the recent advent of early diagnosis.
